# Impact of Cholesterol on Ischemic Stroke in Different Human-Like Hamster Models: A New Animal Model for Ischemic Stroke Study

**DOI:** 10.3390/cells8091028

**Published:** 2019-09-04

**Authors:** Lili Wei, Haozhe Shi, Xiao Lin, Xin Zhang, Yuhui Wang, George Liu, Xunde Xian

**Affiliations:** 1 Key Laboratory of Molecular Cardiovascular Sciences, Institute of Cardiovascular Sciences, Ministry of Education, Peking University, Beijing 100191, China; 2 School of Medicine, Shihezi University, Shihezi City, Xinjiang Uygur Autonomous Region 832000, China; 3 Hebei Invivo Biotech Co, Shijiazhuang 050000, China

**Keywords:** LDLR, ABCA1, gold syrian hamster, ischemic stroke

## Abstract

Rationale: While high low-density lipoprotein cholesterol (LDL-C) and low high-density lipoprotein cholesterol (HDL-C) levels are positively associated with cardiovascular events, it is still unclear whether familial hypercholesterolemia (FH) and Tangier’s disease (TD), caused by mutations in LDLR and ABCA1, respectively, influence ischemic stroke (IS) in humans. Objective: We sought to establish an easier, more effective, and time-saving method to induce IS, then studied the precise effects of different types of lipoproteins on IS. Methods and Results: A new technique termed contralateral middle cerebral artery occlusion (c-MCAO) was introduced to human-like hamster models to induce IS. Compared to traditional distal MCAO (d-MCAO) induced by electrocoagulation, c-MCAO resulted in a more severe IS with larger infarct sizes and more blood–brain barrier (BBB) disruption after 24 h. It was shown that c-MCAO markedly elicited an increase in brain infarct volume and BBB leakage in both homozygous LDLR (LDLR^–/–^) and ABCA1 knockout (ABCA1^–/–^) hamsters, but not in heterozygous LDLR knockout (LDLR^+/–^) hamsters when compared to wild-type (WT) controls. Conclusions: Using human-like genetically engineered hamsters, our findings demonstrated that both high LDL-C level caused by homozygous LDLR deficiency and severe low HDL-C level caused by deleting ABCA1 were risk factors of IS. As such, we believe the development of this novel IS hamster model is suitable for future ischemic/reperfusion studies.

## 1. Introduction

Stroke is a leading cause of death and disability worldwide. Ischemic stroke (IS), accounting for approximately 80% of such cases [1,2], is characterized by the deprivation of blood supply following a thromboebolic occlusion of a major cerebral artery. Accumulative evidence from epidemiological and experimental animal studies suggests that there are many risk factors of ischemic stroke [3]. However, many potential mechanisms and their underlying significance in the pathogenesis of IS are not fully understood.

Cholesterol is a major structural and functional component of cell membranes. It has been reported that elevated plasma cholesterol levels, especially in low density lipoprotein (LDL), are positively associated with the incidence of cardiovascular disease (CVD) [4,5,6,7]. LDL receptor (LDLR) is the most important protein for cholesterol transport. As such, loss-of-function mutations in LDLR, termed “familial hypercholesterolemia (FH)”, lead to a significantly increased serum LDL cholesterol (LDL-C) levels in patients, with cholesterol concentrations ranging from 350 to 500 mg/dL and 650 to 1000 mg/dL in heterozygotes and homozygotes, respectively. Although the relationship of FH and CVD is clear, the influence of FH and elevated cholesterol levels on IS are still controversial [8,9,10,11]. Besides LDL-C, high density lipoprotein cholesterol (HDL-C) had been a promising target in the past few decades because of its roles in reverse cholesterol transport and anti-inflammation, suggesting that plasma HDL-C is negatively associated with CVD [12,13]. To our knowledge, ATP-binding cassette transporter member A1 (ABCA1) is the key protein critical for the modulation of HDL metabolism in both peripheral tissues and the brain [14,15,16]. ABCA1 regulates the efflux of cellular cholesterol to apolipoprotein A-I (ApoA-I). Thus, mutations in ABCA1 results in Tangier disease (TD), which presents with extremely low circulating HDL-C levels [17,18]. However, whether ABCA1 plays a role in IS is limited, and outcomes from different clinical studies are also controversial [19,20]. Taken together, these conflicting results imply that more investigations are needed to elucidate the impact of dyslipidemia on IS. Utilizing suitable human-like experimental animal models, the pathogenesis and development of new potential interventions of IS may be further clarified.

Golden Syrian hamsters exhibit metabolic features similar to humans [21,22,23], suggesting that they could be a highly selective animal model for translational studies in the field of vaso-occlusive disease, especially IS. At present, although middle cerebral artery occlusion (MCAO) had been widely used to create ischemic mice, similar MCAO hamster models have not been reported, with the exception of a single d-MCAO hamster model described by Nagai [24]. Therefore, it would be beneficial to develop an easier and time-saving method to generate an ischemic hamster model that could test our hypothesis that cholesterol in various lipoproteins would differentially affect IS, such as human-like hamsters with LDLR or ABCA1 deficiency generated using CRISPR/Cas9.

## 2. Methods and Materials

### 2.1. Animals

Generation of LDLR knockout hamsters was described previously [25]. The hamster *Abca1* gene was deleted using CRISPR/CAS9 technology, with a gRNA targeting the sequence of CCTGATCCTGATCCGTCCGCCTGAGCTACCCGCCCTATGAACAACATGAGTGAGTG in the third exon, based on gene information (Gene ID: 1018333). Target genome fragments (ABCA1-X-F1: TCAGAGCCCAGCAGCAGGT; ABCA1-X-R1: AGCCAGCCATCACCGAGT) were verified by PCR, and deletion sites were investigated by sequencing. Golden Syrian hamsters with different genotypes were maintained with a 14 h light/10 h dark cycle and fed a standard laboratory chow diet with water ad libitum. Experimental procedures were conducted under the guidelines of Care and Use of Laboratory Animals published by the US National Institutes of Health (No.85Y23, revised 1996). Animal experiments were approved by the Animal Care Committee of Peking University Health Science Center (No. LA2015012). Five-month old male wild-type (WT), LDLR^+/–^, LDLR^–/–^ and ABCA1^–/–^ golden Syrian hamsters were used in the study.

### 2.2. Focal Cerebral Ischemic Model

After acclimatization, hamsters were anesthetized with 3% pentobarbital sodium (45 mg/kg by intraperitoneal injection). Animals underwent MCAO surgery as described by the focal cerebral ischemic model [24].

For distal-MCAO (d-MCAO), a U-shape curvilinear skin incision was performed from the right orbit to the auricle. The exposed temporal fascia and muscle were incised and out-turned. A small opening (2 to 3 mm in diameter) was made in the region of the MCA by a handheld drill perfused with saline to prevent brain injury. The inner layer of the skull and the meninges were removed with forceps and the middle cerebral artery (MCA) was interdicted with the ophthalmobipolar device (Shanghai Medical Devices Co., Ltd., Shanghai, CHN, China), and then transected distally. Finally, the temporal muscle and skin were sutured back in place. Thereafter, animals were placed in a supine position, and had both common carotid arteries (CCA) exposed. The right CCA was ligated with a 6-0 nylon thread. The left CCA was occluded by the nylon thread with a slipknot for 30 min, then removed to restore blood circulation, and the skin was sutured. Rectal temperature of the hamster was maintained at 37 ± 0.5 °C with a heating pad (Harvard Apparatus, Holliston, MA) until the animals woke up and recovered from surgery. The hamsters were returned to their cages and had their physical condition monitored for several hours continuously.

In our study, we developed a new method of MCA occlusion by inserting a monofilament into the external carotid artery (ECA) unilaterally to block blood flow to the middle cerebral artery, and simultaneously ligating the contralateral common carotid artery for 40 min, for which we termed contralateral-MCAO (c-MCAO). We made a 2 cm long midline incision on the neck to gently separate soft tissues under a stereo microscope, then dissected the right CCA and ECA, isolating the internal carotid artery (ICA) from the surrounding structures and then placing a tight slipknot on the CCA and ICA to block the blood flow with a 6-0 silk suture to temporarily reduce the arterial pressure. A dead knot with a silk suture was then applied to the ECA and then cauterized. A suture loop was placed at the ECA stump near the ICA bifurcation and a small hole was cut in the vessel wall of the ECA stump, followed by the introduction of a silicon rubber-coated mono-filament (Doccol Corp, Redlands, CA, USA) into the ECA lumen down towards the bifurcation, which was used to gently pull back the ECA stump to allow the filament to slide into the ICA and then tighten the suture loop at the ECA stump to secure the filament. We then unfastened the slipknot at the ICA and the filament inside the ICA lumen was tied tightly. The left CCA was subsequently dissected and temporarily ligated for 40 min. Ischemia was confirmed by monitoring regional cerebral blood flow (CBF) in the area of the right MCA with a laser doppler transducer.

### 2.3. Analysis of Plasma Lipids in Hamsters on Chow Diet

Plasma was collected from WT, LDLR^+/–^, LDLR^–/–^, ABCA1^–/–^ hamsters after 12 h fasting. Triglyceride (TG) and total cholesterol (TC) were determined using commercial enzyme-catalyzed kits (Sigma, TR0100-1KT, MAK043-1KT; Applygen, E1006, respectively). HDL-C levels were measured with a TC kit after precipitating apoB-containing lipoproteins by 20% polyethylene glycol (PEG). Pooled plasma aliquots (100 μL) were used to analyze lipoprotein profiles by fast-protein liquid chromatography (FPLC, Tricorn high-performance Superose S-6 10/300GL column) at a flow rate of 0.5 mL/min. The mobile phase buffer with pH 7.5 contains NaCl (0.15 mol/L), Na_2_HPO_4_ (0.01 mol/L), and EDTA (0.1 mmol/L). The eluted fractions (500 μL per fraction) were assayed for cholesterol content shown as micro gram per fraction (μg/fraction). In the following experiment, all hamsters were tested 24 h after operation.

### 2.4. Infarct Volume Quantification

Brains were collected and sliced into 1 mm thick sections, and then incubated in 1% TTC (2, 3, 5-triphenyltetrazolium chloride) solution for 15 min at 37 °C. Infarcted areas were photographed as described [26] for the determination of infarction volumes and edema, measurements were corrected with image analysis software (ImagePro Plus, OLYMPUS Shinjuku-ku, Tokyo, Japan).

### 2.5. Blood–Brain Barrier (BBB) Permeability

BBB permeability was measured according to a previously reported procedure [27]. Briefly, 2% Evan’s blue (EB) dye (Sigma) was injected intravenously into the venae sublingualis at a dose of 4 mL/kg and was allowed to circulate for 4 h, followed by PBS perfusion via the left cardiac ventricle. Then the brains were harvested, homogenized in 500 μL of PBS, and centrifuged with 15,000 rcf at 4 °C for 30 min. The resultant supernatant was collected, and an equal volume of 50% trichloroacetic acid was added, followed by the centrifugation. The concentration of Evan’s blue dye was measured by a spectrophotometer and calculated according to a standard curve (Genesis 10 µV; Thermo Fisher Scientific, Waltham, MA, USA).

### 2.6. Immunohistochemistry

At the end point of our experiments, hamsters were sacrificed and perfused with 0.01 M cold phosphate buffered solution (PBS), followed by 4% paraformaldehyde (PFA) solution through the left ventricle. The brains were harvested, fixed in 4% (PFA) solution for 24 h, and then dehydrated with 20% and 30% sucrose solutions overnight. After fixation, brain tissues were embedded in OCT, cross-sectioned (10 μm per slice), and stored at −20 °C for future use. To characterize the brain lesions, Immunohistochemistry was performed using cryo-sections of the brain with the primary antibodies against CD68 (1:100 rabbit polyclonal IgG; BA3638, BOSTER) or GFAP (1:200 rabbit polyclonal IgG; Ab7260, Abcam) The slices were then incubated with appropriate biotinylated second antibodies (1:200, ABC Vectastain; Vector Laboratories, Burlingame, CA, USA) in 2% normal blocking serum and visualized using 3, 3′-diaminobenzidine (DAB, Vectastain; Vector Laboratories).

### 2.7. Quantitative Real-Time PCR

Total RNA was extracted from whole brain tissues by the reagent Trizol (Invitrogen, Carlsbad, CA, USA, 12183555), and the first-strand cDNA was reversely transcribed using a RT kit (Invitrogen, 18091200). All amplification reactions were performed using the Mx3000 Multiplex Quantitative PCR System with 40 cycles of 95 °C for 30 s, 60 °C for 30 s, and 72 °C for 30 s. The CT values normalized to the internal control 18S were used to evaluate gene expression. Results were presented as a ratio of values compared to WT.

### 2.8. Hematologic Parameters

Blood was collected from the retro-orbital plexus in the animals before c-MCAO surgery. Twenty μL of fresh blood samples were used for the measurement of the following hematologic parameters: Red blood cell count (RBC), white blood cell count (WBC), hemoglobin (HGB), hematocrit (HCT), mean corpuscular volume (MCV), mean corpuscular hemoglobin (MCH), mean corpuscular hemoglobin concentration (MCHC), platelet count (PLT), mean platelet volume (MPV), platelet distribution width (PDW), lymphocyte count (LYM), and lymphocyte percentage (LYM%).

### 2.9. Statistical Methods

All data were expressed as mean and standard deviation (mean ± SD). Statistical significance analysis was performed using SPSS 21.0 for Windows (SPSS Inc., Chicago, IL, USA) with Student’s *t*-test or one-way ANOVA. A value of *p* < 0.05 was considered to be statistically significant.

## 3. Results

### 3.1. c-MCAO Significantly Increased the Ischemic Stroke Severity

**Figure 1:**
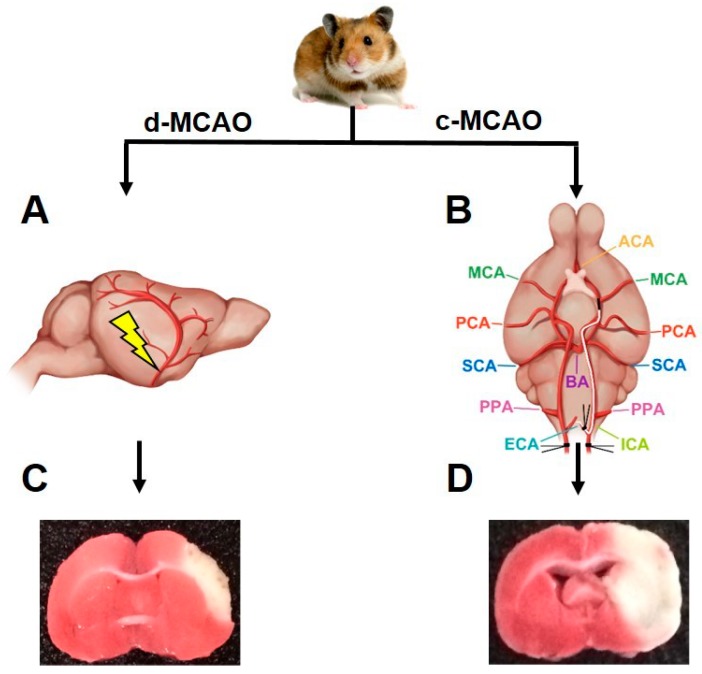
Representative diagram of cerebral ischemic hamster models using distal middle cerebral artery occlusion (d-MCAO) and contralateral middle cerebral artery occlusion (c-MCAO). (**A**) Model representing d-MCAO, in which blood flow is blocked by electrocoagulating the distal middle cerebral artery. (**B**) Model representing c-MCAO, in which a silicon-coated intraluminal suture occludes the origin of the MCA. The right external carotid artery (ECA) is ligated and cauterized to a stump, where the suture goes through into the artery from internal carotid artery (ICA) to MCA, simultaneous ligation of the contralateral CCA is performed for 40 min. ACA, anterior cerebral artery; PCA, posterior cerebral artery; SCA, superior cerebellar artery; BA, basilar artery; PPA, pterygopalatine artery. (**C**,**D**) 2, 3, 5-triphenyltetrazolium chloride (TTC) staining; white area indicates the infarct and red area is the normal tissue.

**Figure 2:**
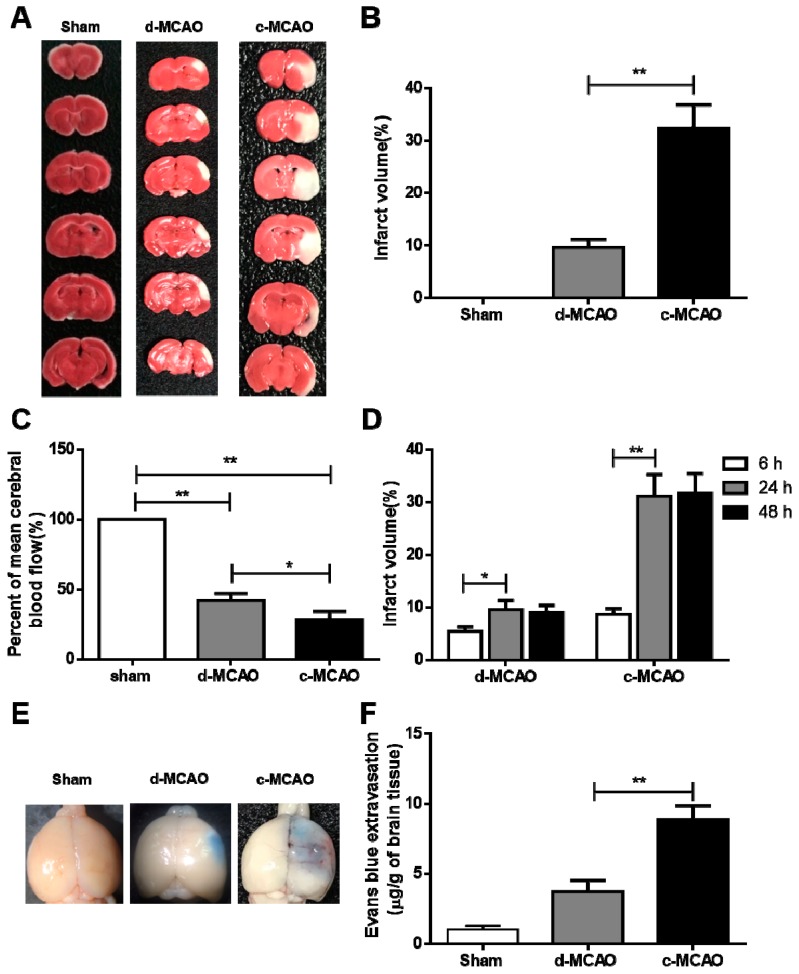
Comparison of the severity of ischemic strokes induced by d-MCAO and c-MCAO. Wild-type (WT) hamsters were subjected to d-MCAO and c-MCAO for 24 h.(**A**) Representative images of six coronal brain sections stained with 2, 3, 5-triphenyltetrazolium chloride (TTC) staining. (**B**) Quantification of the infarct volumes in Sham, d-MCAO, and c-MCAO from (**A**) (*n* = 6/group). (**C**) Measurement of cerebral blood flow after ischemia by laser Doppler transducer (*n* = 6–8/group). (**D**) Time course analysis of infarct volume in ischemic hamsters induced by d-MCAO and c-MCAO (*n* = 6–8/group). (**E**) Representative images of the Evan’s blue (EB)-stained whole brains in sham, d-MCAO, and c-MCAO group. (**F**) Quantification of the Evan’s blue extravasation; data are expressed as mean ± SD. * *p* < 0.05; ** *p* < 0.01 versus sham group.

**Figure 3:**
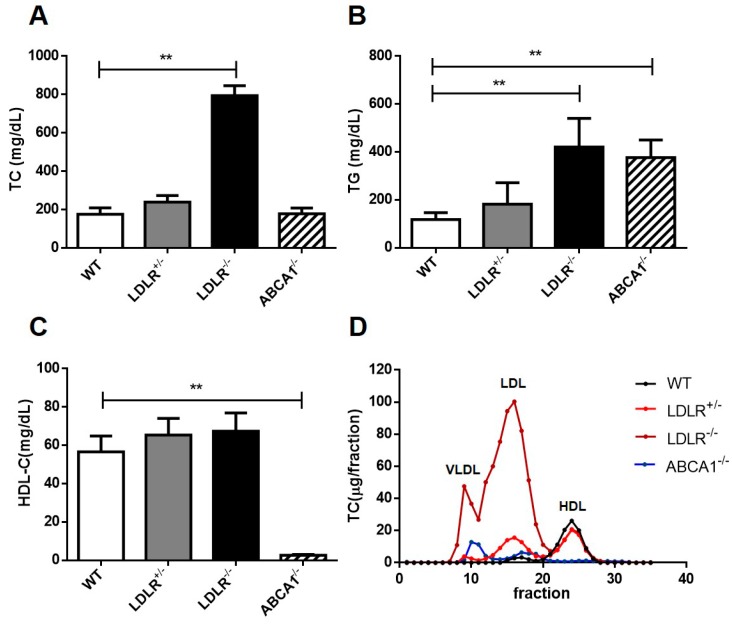
Analysis of plasma lipid profiles in different ischemic hamster models fed on chow diet. (**A–C**) Plasma total cholesterol (TC) (**A**), triglyceride (TG) (**B**) and high-density lipoprotein cholesterol (HDL-C) (**C**) were determined in male WT, LDLR^+/–^, LDLR^–/–^ and ABCA1^–/–^ hamsters after 12 h fasting (*n* = 8/group). (**D**) Fast-protein liquid chromatography (FPLC) analysis of the lipoprotein distribution in pooled plasma from different groups (*n* = 6/group). Data are expressed as mean ± SD. * *p* < 0.05; ** *p* < 0.01 versus WT group.

To establish an ischemic hamster model, we introduced a novel method to WT hamsters based on the classic MCAO method described by the sketch in Figure 1. TTC staining showed that no infarction was observed in the sham group; however, infarction occurred in the two surgical groups, in which white areas were observed in the infarcted brain tissue (Figure 2A). Compared to the d-MCAO model, the infarct size of the c-MCAO model was increased by three times (32.3 ± 2.0% vs 9.6 ± 0.7%). Measurements of CBF by Doppler blood flow meter showed that compared to sham group, both d-MCAO and c-MCAO groups displayed a significant reduction in CBF with 37.9 ± 2.3% and 28.6 ± 2.6%, respectively, indicating that blood flow was much lower and more severely obstructed in the c-MCAO group. In the time progression study, infarct volumes of c-MCAO and d-MCAO were significantly increased from 6 h to 24 h (Figure 3D). Moreover, results from intravenous injection of EB showed the amount of dye leaking out of the brain was correlated with the extent of BBB damage (Figure 2E,F), in which the BBB disruption of d-MCAO (3.7 ± 0.4 μg/g) was significantly less than that of c-MCAO (8.9 ± 0.5 μg/g). Due to the larger infarct volume in the c-MCAO group, damage to BBB was more serious, which caused EB penetration into the brain tissue to present in a dispersed pattern, whereas due to the smaller the lesion volume in the d-MCAO group, the EB distribution was restricted in the brain tissue leading to a darker and more localized blue stain. Taken together, our systematic analysis demonstrated that in comparison with d-MCAO reported in literature, the stroke induced by c-MCAO that we established was greater in severity with a larger infarct size and more profound BBB disruption.

### 3.2. Effects of LDL-C and HDL-C on Ischemic Stroke in Different Gene-Manipulated Hamster Models.

Emerging evidence shows that high LDL-C and low HDL-C levels are positively correlated with the incidence of CVD; however, independent epidemiological studies cannot make a clear conclusion on the relationship between high LDL-C/low HDL-C and IS in different populations [28]. Because hamsters have been reported to exhibit metabolic features similar to humans [22], it warranted us to perform our IS study using different hamster models with high LDL-C or low HDL-C levels. Thereby, we introduced four different hamster models in the present study, including WT, heterozygous LDLR KO (LDLR^+/–^), homozygous LDLR KO (LDLR^–/–^), and ABCA1 KO (ABCA1^–/–^) hamsters. On regular chow diet, LDLR^+/–^ and LDLR^–/–^ hamsters displayed modest and severe hypercholesterolemia, respectively, which was similar to clinical features of HeFH and HoFH patients showing modestly and markedly elevated plasma TC concentrations, respectively (Figure 3A). Moreover, ABCA1^–/–^ hamsters showed phenotypes resembling those with Tangier’s disease (TD) and familial high-density lipoprotein deficiency (FHD) in humans (Figure 3C). Interestingly, both LDLR^–/–^ and ABCA1^–/–^ hamsters also showed increased TG levels when compared to WT (Figure 3B), suggesting that dysregulation of cholesterol elicited the abnormality of TG metabolism that has been reported in TD and FHD patients. Further analysis by FPLC revealed WT hamsters had a distinct LDL peak, which was more pronounced in the LDLR^+/−^ hamsters, whereas LDLR^−/−^ hamsters displayed disproportionately elevated cholesterol levels in the LDL fraction (Figure 3D). Likewise, cholesterol concentration in HDL fraction was almost absent in ABCA1^–/–^ hamsters. Collected data implied that LDLR^–/–^ and ABCA1^–/–^ hamsters on a chow diet developed abnormal lipoprotein profiles similar to those of patients with FH and TD, suggesting that these gene-manipulated hamsters are ideal models for studying the effects of different cholesterol subtypes on IS.

**Figure 4:**
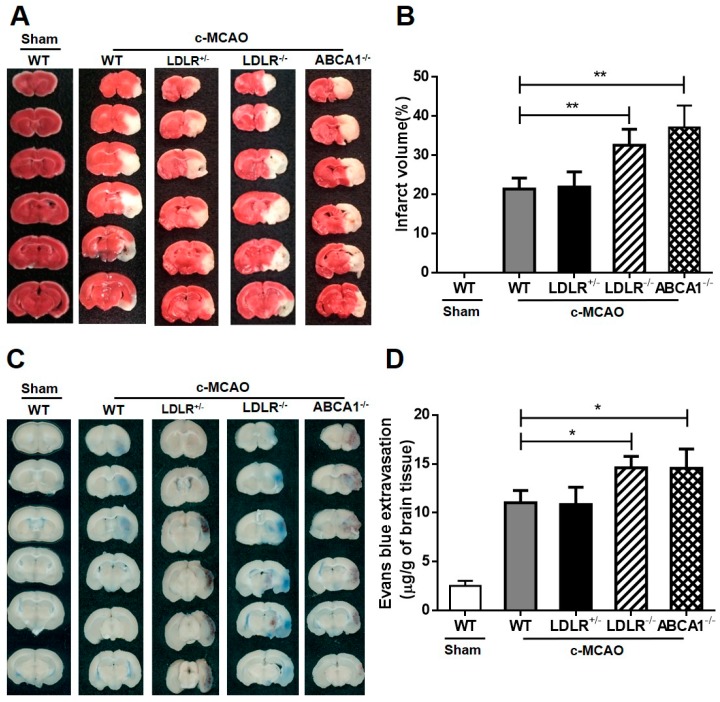
Effects of different types of dyslipidemia on infarct volume and blood–brain barrier (BBB) destruction. (**A**) Representative images of six coronal brain slices showing the cerebral infarct with TTC staining after c-MCAO surgery. (**B**) The infarct volumes of each group were quantified (*n* = 5/group). (**C**) Representative images of the EB-stained brain sections; (**D**) Quantification of the Evan’s blue extravasation (*n* = 5/group); Data are expressed as mean ± SD. * *p* < 0.05; ** *p* < 0.01 versus sham group.

Next, we conducted c-MCAO surgery on four different hamster models and found that both LDLR and ABCA1 knockouts exhibited larger infarct volumes and more EB leakage compared to WT (Figure 4A–D), demonstrating that high LDL-C or low HDL-C could exacerbate ischemic stroke.

### 3.3. Enhanced Brain Inflammation in LDLR^–/–^ and ABCA1^–/–^ Hamsters with IS.

**Figure 5:**
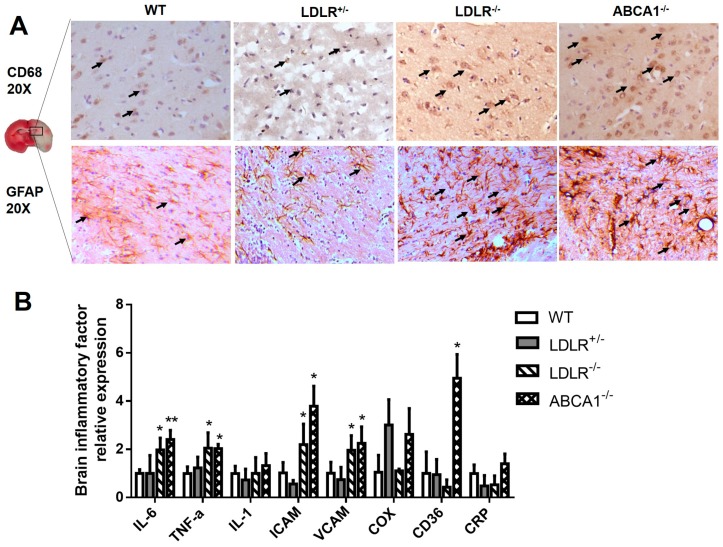
Effect of high LDL-C and low HDL-C on brain inflammation in hamsters with IS. (**A**) Representative images of immunostaining of CD68 and GFAP in ischemic cerebral areas of different genotypes. The arrows indicate positive staining. (**B**) The brain mRNA expression of inflammatory biomarkers in different hamster genotypes (*n* = 8/group). Data are expressed as mean ± SD. * *p* < 0.05; ** *p* < 0.01 versus WT group.

To explore the inflammatory mechanism underlying the pathogenesis of IS in our dyslipidemic hamster models, we investigated the expression of CD68 and GFAP, two markers of cerebral inflammation, by immunohistochemistry. Our results showed that the infiltration of CD68-positive macrophages into the ischemic brain was significantly increased in LDLR^–/–^ and ABCA1^–/–^ hamsters compared to WT and LDLR^+/–^ hamsters (Figure 5A. Upper panel.). Likewise, the expression level of GFAP in astrocytes was also strongly upregulated, implying a local inflammatory response (Figure 5A. Bottom panel). To further confirm cerebral inflammation, we also investigated the mRNA expression of inflammatory biomarkers and found that LDLR^–/–^ and ABCA1^–/–^ hamsters displayed a significant increase in IL-6, TNFα, ICAM, and VCAM, with the upregulation of CD36 also observed in the latter (Figure 5B). In addition, data from hematological parameters showed that when compared to WT and LDLR^+/–^ hamsters, LDLR^–/–^ hamsters had a higher RBC and LYM count; however, ABCA1^–/–^ hamsters exhibited a higher WBC and LYM count, but lower numbers of PLT and reduced MPV and PDW, suggesting that changes of cholesterol metabolism diversely affected hematologic parameters, which may contribute to systemic inflammation and could potentially have predictive value for stroke severity (Table 1).

## 4. Discussion

In this study, we described a new method to induce IS and then investigated the effects of high LDL-C or low HDL-C on IS using human-like genetically engineered hamster models. Utilizing c-MCAO, hamsters exhibited IS of greater severity with larger infarct sizes and more BBB disruption compared to the d-MCAO hamster model, which is also consistent with murine results [29,30]. This protocol allows researchers to better study ischemia/reperfusion in animals without need for permanent occlusion and thus could be used conveniently for applications to assess drug efficacy in future IS studies.

Importantly, our study showed that heterozygous LDLR KO hamsters with modestly elevated plasma TC and LDL-C levels did not display accelerated IS compared to WT animals. This observation was consistent with the outcome from a Copenhagen general population study, in which Beheshti and his colleagues reported that heterozygous FH and high LDL-C did not confer an increased risk for IS [8]. However, this conclusion is incomplete because homozygous FH individuals with higher LDL-C levels were not recruited in the study. Therefore, to investigate whether the homozygous form of FH with hypercholesterolemia influenced the severity of IS, we also performed c-MCAO on homozygous LDLR KO hamsters and found that unlike heterozygous FH in hamsters, homozygous FH showed a significant increase in infarct volume and BBB disruption accompanied by a local inflammatory response, indicating that one copy of the *Ldlr* gene may be sufficient to confer protection against IS, whereas a total loss of the *Ldlr* gene exacerbates IS.

In contrast with FH, where both heterozygous and homozygous forms showed an increase in plasma cholesterol (especially LDL-C, though to different extents), loss-of-function mutations in the *Abca1* gene showed more diverse manifestations of HDL-C levels [31]. In our study, we only used homozygous ABCA1 KO hamsters to mimic clinical TD patients with a total loss-of-function in the *Abca1* gene. As has been observed in humans with TD, homozygous ABCA1 KO hamsters displayed extremely low HDL-C levels, but without major alterations of LDL-C levels. We reported for the first time that lacking HDL-C due to ABCA1 deficiency exacerbated the development of IS, supporting the concept that low HDL-C levels, at least very low HDL-C levels, are associated with an increased IS severity. This effect is likely attributed to defective systemic inflammation resulting from a lack of HDL-C, which was demonstrated to confer protective effects against inflammation in previous studies [32], further substantiated by the altered hematologic parameters detected in our investigation. Therefore, our study provides strong evidence to support the concept that interaction between metabolic variables such as cholesterol and inflammatory markers plays an important role in IS, which was reported previously [33,34].

It is interesting to note that high LDL-C or low HDL-C levels are always accompanied by an increase in TG levels in humans, including FH and TD patients [35]. Consistent with previously reported observations, we also found that plasma TG concentrations were markedly increased in homozygous LDLR or ABCA1 KO hamsters. Recently, hypertriglyceridemia has been identified as an independent risk factor of CVD [36], but we could not exclude the role of TG in IS at the current stage of our study. However, different large scale clinical trials demonstrate that compared to Statins alone, Statin therapy combined with Ezetimibe, an inhibitor of cholesterol absorption at small intestine brush border cells, significantly reduced TG levels and prevented IS [37,38], implying that elevated TG level should still be considered as a risk factor of IS. It would be tempting to study the effects of TG on IS using genetically engineered hamster models with hypertriglyceridemia in the future.

In summary, we employed the new method of c-MCAO to create an IS hamster model, which exhibits metabolic features similar to humans and will be suitable for translational studies of human IS. Our study clarifies that high LDL-C in homozygous FH or low HDL-C in homozygous TD, both accompanied elevated circulating TG, are associated with an increased IS severity, providing new insights into more precise roles that cholesterol carried in different lipoproteins play in human IS.

## Figures and Tables

**Table 1:** Overview of the hematologic parameters in WT, LDLR^+/–^, LDLR^–/–^, and ABCA1^–/–^ hamsters before IS procedure.

	RBC (10^12^/L)	WBC (10^9^/L)	HGB (g/L)	HCT (%)	MCV (fl)	MCH (pg)	MCHC (g/L)	PLT (10^9^/L)	MPV (fl)	PDW	LYM	LYM%
WT	6.3 ± 0.7	10.9 ± 1.6	145.6 ± 21.6	43.15 ± 2.1	60.95 ± 3.1	20.9 ± 1.6	359 ± 44.6	343 ± 129	4.8 ± 1.7	20.9 ± 0.5	2.9 ± 0.8	36.9 ± 4.1
LDLR^+/–^	7.1 ± 0.4	9.7 ± 1.1	140.8 ± 6.7	41.3 ± 1.6	61.8 ± 2.4	19.9 ± 2.1	326.3 ± 39	456.7 ± 32.5	3.4 ± 0.3	17.6 ± 0.6	5.5 ± 1.7	33.5 ± 2.8
LDLR^–/–^	8.3 ± 0.7 *	11.9 ± 1.57	153.7 ± 17.4	41.1 ± 1.5	59.9 ± 2.9	21.5 ± 1.8	344.8 ± 15.5	432.2 ± 18.7	3.4 ± 0.3	17.6 ± 0.5	6.3 ± 1.8 *	52.7 ± 2.5 *
ABCA1^–/–^	7.8 ± 0.3	22.8 ± 2.4 **	167.3 ± 3.7	42.4 ± 0.9	55.9 ± 3.4	20.3 ± 1.0	413 ± 20.3	239.8 ± 33.7 **	1.53 ± 0.3 **	14.3 ± 0.3 **	6.1 ± 1.9*	51.8 ± 1.9 **

Data are expressed as mean ± SD. * *p* < 0.05; ** *p* < 0.01 versus WT. *n* = 8 per genotype.
